# Persistence in the passion investment market^☆^

**DOI:** 10.1016/j.heliyon.2022.e12555

**Published:** 2022-12-22

**Authors:** Guglielmo Maria Caporale, Luis Gil-Alana, Alex Plastun, Ahniia Havrylina

**Affiliations:** a Brunel University London, Uxbridge, United Kingdom; b University of Navarra and Universidad Francisco de Vitoria, Madrid, Spain; c Sumy State University, Sumy, Ukraine

**Keywords:** Passion investment, Persistence, Long memory, R/S analysis, Fractional integration

## Abstract

Research on the passion investment market is still limited, especially on the issue of persistence. This paper is the first to investigate it using both R/S analysis and fractional integration in the case of 3 fine wine price indices, 10 diamond price indices, 15 art price indices, and 1 stamp price index at the daily, monthly and quarterly frequency. The results can be summarised as follows: wine prices are found to be highly persistent, whilst stamp prices appear to be only weakly persistent, though they can still be characterised as a long-memory process; as for diamond prices, they can be persistent (Diamonds & Gems), anti-persistent (Diamonds Carat indices) or even random (Polished Prices Diamond Index). The dynamic R/S analysis also shows that persistence is time-varying and tends to fluctuate around the average. These findings can be explained by the different degree of liquidity of the assets examined and have implications for both academics investigating market efficiency and practitioners interested in forecasting prices.

## 1 Introduction

“Passion investing” in non-traditional assets one is passionate about (such as fine wines, diamonds, stamps, and art objects) has become increasingly popular over the last few decades as an effective way for achieving portfolio diversification. According to Bernales et al. (2020), in 2017 the art and collectibles market attracted more than 35% of high-net-worth individuals. Some of passion collectibles such as diamonds and art objects are used as collateral for obtaining funds (Bernales et al., 2020). Besides being useful as a store of value (similarly to gold), they can be considered as “emotional” consumption assets, which makes them play a dual utility role (both as investment and consumption assets) for both passionate investors and speculators; these have fluctuating tastes and enthusiasm for such assets as well as heterogeneous beliefs resulting in large forecast dispersion for the expected value of the ‘emotional’ income.

Compared to conventional assets, emotional or passionate ones are also more likely to be constrained by limited supply and are generally characterised by higher transaction costs, lower liquidity, informational asymmetry (e.g., insiders such as auction houses often have access to more relevant information), and market inefficiencies accentuated by the design of the auction trading system (Ashenfelter and Graddy, 2011) and the difficulty to apply a short selling strategy. As argued by Aye et al. (2018), the ability of investors in the art market to earn abnormal returns by exploiting predictable price patterns points to the inefficiency of this market; in this respect passionate assets do not differ from conventional ones, for which there is plenty of evidence of persistence in returns and their volatility (Caporale et al., 2019), price bubbles (Scherbina and Schlusche, 2014) and of various types of market anomalies such as calendar ones (Plastun et al., 2019), all suggesting that the Efficient Market Hypothesis (EMH – Fama, 1970) might not hold empirically.

Various studies on passion assets have recently been carried out to examine, for instance, their performance relative to conventional assets (Veld and Veld-Merkoulova, 2007 for stamps; Dimson and Spaenjers, 2014 for stamps and art objects); the diversification benefits of purchasing fine wines (Chu, 2014; Bouri, 2014, 2015; Jurevičienė and Jakavonytė, 2015; Bouri et al., 2018) and diamonds (D'Ecclesia and Jotanovic, 2018; Barbi et al., 2020); the (in)efficiency of the fine wine market (e.g., Bouri et al., 2017; Fernandez-Perez et al., 2019; Masset and Henderson, 2010). Another property of the data property which is interesting to investigate is their degree of persistence, since this can shed light on the issue of market efficiency (Nazlioglu et al., 2022). In the case of passion asset prices, mixed results have been obtained by previous studies. For instance, Goetzmann (1995) applied a Repeat-Sales Regression method to painting sales data from 1716 to 1986 and found evidence of persistence; Chong et al. (2012) analysed the diamonds market using R/S analysis and did not find long memory; Auer (2014) allowed for time variation and concluded that there is negative correlation at the weekly frequency in the diamond market (see also Auer and Schuhmacher, 2013); Munteanu and Pece (2015) also used the R/S method as well as others to analyse persistence in the art market and found persistence in 3 cases out of 4; Aye et al. (2017) examined art price indices using fractional integration methods and detected randomness and persistence in assets characterised by high and low liquidity respectively; Bouri et al. (2017) carried out unit root tests and concluded that the wine market is inefficient (see also Le Fur, 2020; and Kumar, 2021).

Such differences in the results can be explained by the different methods and price series used as well as the different periods considered. The present paper aims to provide more thorough evidence on persistence in the passion market by examining a wide range of price indices (more precisely, 3 for fine wine prices, 10 for diamond prices, 15 for art prices, and 1 for stamp prices) at the daily, monthly and quarterly frequency using two different long-memory methods, specifically R/S analysis and fractional integration. Ours is the most comprehensive evidence to date on the issue of persistence in the passion investment market; it is obtained by exploring different segments of this market (as many as 29 different assets), using different methodologies and carrying out the analysis at different data frequencies, in both cases as a robustness check. This enables us to produce much more reliable results than those reported in previous studies affected by data snooping and other limitations.

The layout of the paper is the following. Section 2 provides a brief review of the literature concerning the issue of persistence in different financial markets and the passion investment market in particular. Section 3 describes the data and outlines the empirical methodology, which includes both R/S analysis and fractional integration methods. Section 4 presents the empirical results and provides some economic interpretation. Section 5 offers some concluding remarks, in particular highlighting the contributions of the paper together with its limitations.

## 2 Literature review

Persistence is an important property of the data which has been extensively investigated. For example, Chin (2022) analysed the dynamics of US inflation persistence and its implications for monetary policy. Numerous studies have focused on persistence in a variety of financial markets. For instance, Baillie et al. (2007) found long memory in daily futures returns for commodities (gold, gasoline, cattle, hogs, soybeans, corn). Volatility persistence was detected by Jin and Frechette (2004) and Sephton (2009) in the case of agricultural future prices, and by Naeem et al., (2019) for gold and silver returns. Gil-Alana et al. (2015) found persistence in gold prices. Evidence of long memory was also provided by Khuntia and Pattanayak (2018) and Caporale et al. (2018) in the case of daily Bitcoin returns, and by Zhou and Kang (2011), Zhou (2016), Liu et al. (2019) for REITs (Real estate investment trusts). Persistence analysis helps to model price dynamics in the financial markets (Boubaker et al., 2022) and to assess market efficiency (Memon et al., 2022).

Analysing persistence is particularly interesting in the case of the passion market, which is likely to be characterised by lower efficiency than other financial markets owing to higher information asymmetry, difficulties with the valuation of the assets reflecting disagreements between the buyer and the seller (Bernales et al., 2020), higher transaction costs and lower trading volumes, high entry barriers and investment risks (Fischer and Firer, 1985), and difficulties in implementing short selling strategies. Thus, a number of studies on this topic have been conducted.

Goetzmann (1995) analysed painting sales data from 1716 to1986 and found that decennial returns exhibit persistence, possibly because of their correlation with inflation, which is positively autocorrelated. Aye et al. (2017) examined long memory in 15 art price indices using fractional integration methods that account for long memory; his findings imply market efficiency only for a few cases characterised by high liquidity (Contemporary and US markets), globalisation, improved flow of information, and a high number of participants. Persistent price behaviour and market inefficiency in the art market was also reported by Assaf et al. (2021). Munteanu and Pece (2015) analysed the stock prices of the four main auction houses and concluded that three of them exhibit persistence and one anti-persistence. Volatility persistency was found by Ameur and Fur (2019).

Chong et al. (2012) investigated instead persistence in daily returns and their volatility for diamonds ranging from 0.3 to 3 Carat (from law quality to flawless); their evidence suggests that long memory is present only in the estimated volatility. Similar results were obtained also by Auer (2014) and Auer and Schuhmacher (2013). Persistence in the wine market was found by Bouri and Roubaud (2016), Le Fur (2020) and Kumar (2021); autoregressive properties were reported by Fernandez-Perez et al. (2019), whilst mean reversion was detected by Bouri et al. (2017).

As can be gathered from the above discussion, the existing literature has reached very different conclusions concerning the presence of randomness, persistence or even anti-persistence in the passion investment market. As a result, it is unclear whether or not market efficiency holds (and thus whether or not active investment strategies aiming to “beat” the market would be appropriate), what model specifications would perform best to forecast prices etc. The aim of our study is to shed light on these issues by analysing a comprehensive dataset with two alternative statistical approaches.

## 3 Data and methodology

Our sample includes the following series at the daily, monthly and quarterly frequency: 3 fine wine price indices (Liv-ex Bordeaux 500 Index, Liv-ex Fine Wine 100 Index and Liv-ex Fine Wine Investables Index) over the period 1991–2021; 10 diamond price indices (Diamonds-1 Carat Commercial Index, Diamonds-1 Carat Mixed Index, Diamonds-0.3 Carat Mixed Index, Diamonds-1 Carat Fine Index, Diamonds-0.3 Carat Commercial Index, Diamonds-0.3 Carat Fine Index, Diamonds-0.5 Carat Commercial Index, Diamonds-0.5 Carat Fine Index, Diamonds-0.5 Carat Mixed Index and Polished Prices Diamond Index) over the period 1989–2021 in the case of the first 3 indices, and 2001-2021in the case of the last 7; 15 art price indices (Global Index in USD, Global Index in EUR, Painting, Sculpture, Photography, Drawing, Print, Old Masters, 19th Century, Modern Art, Post-War, Contemporary, USA in USD, UK in GBP and France in EUR) over the period 1998–2021; 1 stamp index (Stanley Gibbons Stamp Index) over the period 1989–2021. The data sources are London International Vintners Exchange (Liv-ex), Fairfield County Diamonds (https://www.diamondse.info/), Artprice (Artprice.com), and the Stanley Gibbons group (ww.stanleygibbons.com/publishing/gibbons-stamp-monthly), respectively.

To evaluate persistence two different methods are applied: R/S analysis (both static and dynamic) and fractional integration. The former is based on the Hurst exponent (H) which is the measure of persistence lying in the interval [0, 1]. Persistence is found when H > 0.5. Random data are characterised instead by H = 0.5. Anti-persistence is detected when H < 0.5.

The Hurst exponent *H* is the estimated slope coefficient in the following equation: *log (R/S) = log (c) + H∗log (n)* (Hurst, 1951). More precisely, the estimation procedure is the following:

1. The original data set is transformed into a data set $N_{i}$ consisting of log returns:

. (1) $$N_{i}=\log\left(\frac{Close_{t}}{Close_{t-1}}\right),t=1,2,...\left(M-1\right)$$

2. This data set is divided into contiguous *A* sub-data sets with length n, such that *A*_*n*_ *= N*, then each sub-data set is identified as Ia, given the fact that a = 1, 2, 3. . ., A. Each element Ia is represented as Nk with k = 1, 2, 3. . ., N. For each Ia with length n the average $e_{a}$ is defined as:

(2) $$e_{a}=\frac{1}{n}\sum_{k=1}^{n}N_{k,a},k=1,2,3,...N,а=1,2,3...,А\text{.}$$

3. Accumulated deviations *X*_*k,a*_ from the average $e_{a}$ for each sub-period *I*_*a*_ are calculated as:

(3) $$X_{k,a}=\sum_{i=1}^{k}\left(N_{i,a}-e_{a}\right)\text{.}$$

The range is defined as the maximum index *X*_*k,a*_ minus the minimum *X*_*k,a*_, within each sub-period (*I*_*a*_):

(4) $$R_{Ia}=\max\left(X_{k,a}\right)-\min\left(X_{k,a}\right),1\leq k\leq n\text{.}$$

4. The standard deviation $S_{Ia}$ is calculated for each sub-period *I*_*a*_:

(5) $$S_{Ia}={\left(\left(\frac{1}{n}\right)\sum_{k=1}^{n}{\left(N_{k,a}-e_{a}\right)}^{2}\right)}^{0,5}\text{.}$$

5. Each range *R*_*Ia*_ is normalised by dividing by the corresponding *S*_*Ia*_. Therefore, the re-normalised scale during each sub-period *I*_*a*_ is *R*_*Ia*_*/S*_*Ia*_. In step 2 above, adjacent sub-data sets of length n are obtained. Thus, the average R/S for length *n* is defined as:

(6) $${\left(R/S\right)}_{n}=\left(1/A\right)\sum_{i=1}^{A}\left(R_{Ia}/S_{Ia}\right)$$

6. The length *n* is increased to the next higher level *(M - 1)/n*, and must be an integer number. In this case, *n*-indices that include the start and end points of the time series are used, and Steps 1–6 are repeated until *n = (M - 1)/2.*
7. Next one can use least squares to estimate the equation *log (R/S) = log (c) + Hlog (n).* The slope coefficient in this regression is an estimate of the Hurst exponent *H.*

To perform dynamic R/S analysis a sliding-window approach is used (see Caporale et al., 2016 for more details). Specifically, the Hurst exponent is calculated using a data window based on a given number of observations (300 in the present case) which is shifted various times till reaching the end of the sample, the size of the shift being 50 (Caporale et al., 2016). For example, for a data set including 1200 observations there will be 18 shifts ((1200–300)/50) and 19 estimates of the Hurst exponent will be obtained.

It should be mentioned that some studies have argued that R/S analysis is biased (Taqqu et al., 1995; Lo, 1991) and have proposed alternative methods such as DFA and DMA (Zhi-Qiang et al., 2019). However, the former is still the commonly used method used to calculate the Hurst exponent in the case of financial data (Mynhardt et al., 2014; Raimundo and Okamoto, 2018; Danylchuk et al., 2020; Metescu, 2022), and it has been shown to perform well by constructing data sets with different properties (randomness, persistence, anti-persistence) to which it has been applied (Caporale et al., 2016).

The second method employs I(d) techniques to measure persistence as the differencing parameter d which is related to the Hurst exponent described above through the relationship H = d + 0.5. Note, however, that we conduct the R/S analysis for the return series (the first differences of the logged indices), while I(d) models are estimated for the logged indices themselves, in which case the relationship becomes H = (d – 1) + 0.5 = d – 0.5. We consider processes of the following form:

(7) $${\left(1-B\right)}^{d}x_{t}=u_{t},t=1,2,...,$$

where B is the backshift operator (Bx_t_ = x_t-1_); u_t_ is an I (0) process (which may incorporate weak autocorrelation of the AR (MA) form) and x_t_ stands for the errors of a regression model of the form:

(8) $$y_{t}=\beta_{0}+\beta_{1}t+x_{t},t=1,2,...,$$

where y_t_ denotes the log of the stock index in each case, β_0_ and β_1_ denote the constant and the coefficient on a linear time trend t to be estimated, and the regression errors x_t_ are I(d). Note that under the Efficient Market Hypothesis the value of d in (7) should be equal to 1 and u_t_ should be a white noise process. We use both parametric and semi-parametric methods, in the former case assuming uncorrelated (white noise) error and in the latter autocorrelated errors specified as in Bloomfield (1973). More specifically, we use the Whittle estimator of d in the frequency domain (Dahlhaus, 1989; Robinson, 1994, 1995), as described, for example, in Gil-Alana and Robinson (1997).

## 4 Empirical results

The static Hurst exponent for the Wine and Stamp indices is reported in Table 1.

**Table 1:** Static Hurst exponent calculations for the Wine and conventional Stamp indices.

Type	Instrument	Hurst exponent
Wine	Liv-ex Bordeaux 500 Index	0.78
	Liv-ex Fine Wine 100 Index	0.85
	Liv-ex Fine Wine Investables Index	0.78
Stamps	STANLEY GIBBONS GROUP	0.59

The high values in the case of wine prices provide evidence of both persistence and long memory in these series. More specifically, the estimated Hurst exponent in the range 0.78–0.85 suggests that past prices contain significant information about current and future ones and thus autoregressive models can be used for forecasting purposes in the case of all the analysed wine prices. Stamp prices are less persistent, but still exhibit long memory as the Hurst exponent is much higher than 0.5. The implication is that both sets of prices are predictable (though to a lesser extent in the case of stamp prices), which is inconsistent with the Efficient Market Hypothesis according to which asset prices should follow a random walk.

The static Hurst exponent for the Diamond indices is reported in Table 2. As can be seen, these results are mixed, ranging from persistence (in the case of the Diamonds & Gems) to anti-persistence (for the Diamonds Carat indices) and even randomness (for the Polished Diamond Price index), which possibly reflects different degrees of liquidity.

**Table 2:** Static Hurst exponent calculations for the Diamond indices.

Type	Instrument	Hurst exponent
Diamonds	CCARBNS-DS Diamonds & Gems - PRICE INDEX	0.61
	NORCS-DS Diamonds & Gems - PRICE INDEX	0.60
	WORLD-DS Diamonds & Gems - PRICE INDEX	0.60
	Diamonds-1 Carat Commercial Index - PRICE INDEX	0.35
	Diamonds-1 Carat Mixed Index - PRICE INDEX	0.45
	Diamonds-0.3 Carat Mixed Index - PRICE INDEX	0.46
	Diamonds-1 Carat Fine Index - PRICE INDEX	0.36
	Diamonds-0.3 Carat Commercial Index - PRICE INDEX	0.40
	Diamonds-0.3 Carat Fine Index - PRICE INDEX	0.32
	Diamonds-0.5 Carat Commercial Index - PRICE INDEX	0.38
	Diamonds-0.5 Carat Fine Index - PRICE INDEX	0.31
	Diamonds-0.5 Carat Mixed Index - PRICE INDEX	0.42
	Polished Prices Diamond Index - PRICE INDEX	0.51

In particular, the Diamonds & Gems price series have a Hurst exponent significantly above 0.5 and thus exhibit long memory and are predictable using past values. By contrast, the Polished Diamond Price index data have a Hurst exponent close to 0.5, which does not suggest either long memory or predictability. Finally, the Diamonds Carat indices have a Hurst exponent below 0.5, which indicates that price reversals occur, namely falls should be expected after rises and vice versa.

The different results for different types of diamonds may reflect differences in the degree of liquidity and in trading volumes.

**Figure 1:**
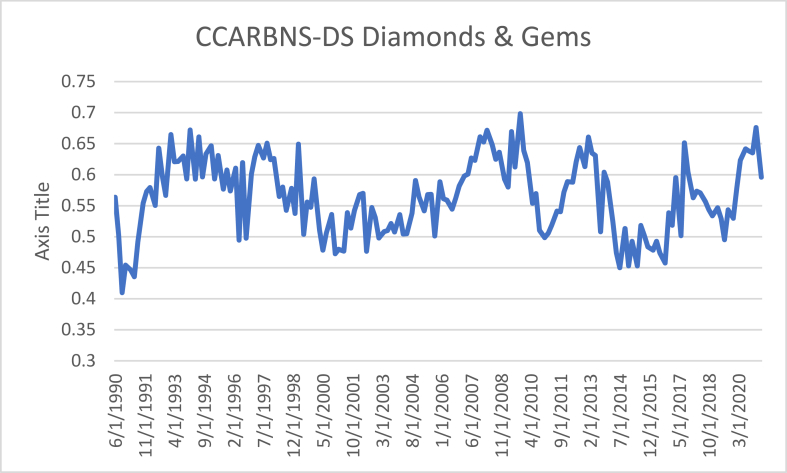
Dynamic Hurst exponent calculations for the case of CCARBNS-DS Diamonds & Gems.

The next step is the dynamic R/S analysis, which provides information about changes in persistence over time. The results are plotted in Figure 1. Visual inspection suggests that persistence is time-varying and tend to fluctuate around its average.

Figure 1 provides clear evidence that persistence is unstable over time; its range is 0.45–0.70, which means that some periods might be characterised by anti-persistence or even randomness, which might explain the different results in Table 2.

The static Hurst exponent for the Art price indices is reported in Table 3.

**Table 3:** Static Hurst exponent calculations for the Art price indices.

Type	Instrument	Hurst exponent
Artprice	Global Index (USD)	0.43
	Global Index (EUR)	0.39
	Painting	0.54
	Sculpture	0.57
	Photography	0.33
	Drawing	0.65
	Print	0.50
	Old Masters	0.36
	19th Century	0.39
	Modern Art	0.53
	Post-War	0.50
	Contemporary	0.37
	USA (USD)	0.54
	UK (GBP)	0.35
	France (EUR)	0.61

Different assets within the art price category are characterized by different persistence properties. Post-War and Print have a Hurst exponent of 0.5 and thus do not exhibit long memory and can be characterised as random. By contrast, Drawing, Sculpture and France (EUR) prices have a Hurst exponent significantly above 0.5, which implies long memory properties. Most assets in the art price category have a Hurst exponent much below 0.5 and thus are characterised by anti-persistence.

As can be seen, there is again a wide range of results, which could reflect differences between categories of art objects (e.g., photography and sculpture), the currencies used (with different degrees of persistence influencing art prices) and the specific point in time considered. A summary is presented in Table 4, where indices are grouped by their degree of persistence.

**Table 4:** Summary of the results for the static Hurst exponent.

Anti-persistent	Random	Persistent
<0.45	0.45–0.55	>0.55
• Diamonds-1 Carat Commercial Index • Diamonds-1 Carat Mixed Index • Diamonds-1 Carat Fine Index • Diamonds-0.3 Carat Commercial • Diamonds-0.3 Carat Fine Index • Diamonds-0.5 Carat Commercial • Diamonds-0.5 Carat Fine Index • Diamonds-0.5 Carat Mixed Index • Artprice Global Index (USD) • Artprice Global Index (EUR) • Photography • Old Masters • 19th Century • Contemporary • Artprice UK (GBP)	• Diamonds-0.3 Carat Mixed Index • Polished Prices Diamond Index • Painting • Print • Modern Art • Artprice Post-War • Artprice USA (USD)	• Liv-ex Bordeaux 500 Index • Liv-ex Fine Wine 100 Index • Liv-ex Fine Wine Investables Index • CCARBNS-DS Diamonds & Gems • NORCS-DS Diamonds & Gems • STANLEY GIBBONS GROUP • Sculpture • Drawing • Artprice France (EUR)

Persistence implies predictability (and therefore represents evidence of market inefficiency), which suggests that autoregressive models can be used to predict prices, whilst anti-persistence indicates that the series revert to their mean more often than a random series would. To shed further light on these issues we report in Table 5 partial correlation functions with the corresponding t-statistics and p-values for all the indices under investigation. p-values below 0.05 indicate that the corresponding lag length is the appropriate one for the autoregressive model to be used for the data under examination.

**Table 5:** Partial autocorrelations: the case of Liv-ex Fine Wine Investables Index, Diamonds-0.5 Carat Fine Index and Diamonds-0.5 Carat Fine Index.

Time lag k	Liv-ex Fine Wine Investables Index (persistent)			Diamonds-0.5 Carat Fine Index (anti-persistent)			Polished Prices Diamond Index (random)		
	PACF	T-STAT	P-value	PACF	T-STAT	P-value	PACF	T-STAT	P-value
1	0.99	18.86	0.00	0.97	69.71	0.00	0.99	70.72	0.00
2	-0.06	-1.15	0.13	0.23	16.76	0.00	0.46	32.73	0.00
3	-0.05	-0.88	0.19	0.14	10.36	0.00	0.28	19.82	0.00
4	-0.03	-0.55	0.29	0.14	10.10	0.00	0.19	13.36	0.00
5	-0.01	-0.22	0.41	0.09	6.54	0.00	0.74	52.89	0.00
6	-0.01	-0.14	0.44	0.11	7.83	0.00	-0.53	-37.82	0.00
7	-0.02	-0.31	0.38	0.09	6.66	0.00	-0.03	-2.35	0.01
8	-0.01	-0.22	0.41	0.08	5.43	0.00	0.06	4.59	0.00
9	-0.01	-0.26	0.40	0.13	9.51	0.00	0.11	8.06	0.00
10	-0.01	-0.14	0.44	0.11	8.07	0.00	0.29	20.43	0.00

As can be seen, in the case of the persistent series (Liv-ex Fine Wine Investables Index, Hurst exponent = 0.78) p < 0.05 holds only for lag 1, namely this is the appropriate autocorrelation model, whilst for the anti-persistent (Diamonds-0.5 Carat Fine Index, Hurst exponent = 0.31) and random (Polished Prices Diamond Index, Hurst exponent = 0.51) series the p-values for all the first 10 lags are below 0.05, i.e. it is not clear what autocorrelation structure should be preferred, which can be seen as indirect evidence against the presence of long memory in the data, which is indirect evidence against the presence of long memory in the data.

Additional evidence is obtained using I(d) techniques. Specifically, we estimate the model given by Eqs. (7) and (8) and report the results for the two cases of white noise and autocorrelated errors, in the latter case using the exponential spectral model of Bloomfield (1973). This is a non-parametric method to capture autocorrelation implicitly using the spectral density function, and it has been shown to describe well weak dependence in the context of fractional integration (see, e.g., Gil-Alana, 2004). The model is now specified as follows:

(9) $$y_{t}=\beta_{0}+\beta_{1}t+x_{t},{\left(1-B\right)}^{d}x_{t}=u_{t},t=0,1,...,$$

where y_t_ is the observed time series, B is the backshift operator and u_t_ is an I (0) process assumed to be in turn white noise or autocorrelated as in Bloomfield (1973). Note that higher values of d indicate higher persistence but mean reversion occurs as long as d is smaller than 1.

The results based on the assumption of white noise errors are reported in Appendix A for wines and stamps, Appendix B for diamonds, and Appendix C for art prices. More specifically, Tables A1, B1 and C1 report the estimated values of d along with their 95% confidence intervals for the three specifications normally considered in the unit root literature, namely: 1) no deterministic terms, 2) a constant only, and 3) a constant and a linear time trend; the coefficients in bold in these tables are the estimates from the preferred models selected on the basis of the statistical significance of the coefficients on the deterministic terms – these are shown in Tables A.2, B.2 and C.2 together with the corresponding value of d.

It can be seen from Table A.2 that in the case of wines and stamps the time trend is always insignificant and the estimated values of d are much higher than 1, which implies that mean reversion does not occur and thus shocks have permanent effects, i.e. the series are very persistent. In fact, the results even reject the unit root null hypothesis since the confidence intervals include in all cases values which are above 1.

In the case of diamonds (Appendix B, Table B.2) the time trend is negative and significant for six out of the thirteen indices examined, and, in contrast to wine and stamps, mean reversion occurs in most cases, the exceptions being CCARBNS, NORCS and WORLD. The lowest values of d (and thus, the fastest mean reversion in response to shocks) are estimated for Diamonds-1 Carat Fine (0.56) and Diamonds-0.5 Carat Fine (0.58). Thus, most of these series are persistent but mean-reverting, with values of d significantly smaller than 1.

Finally, for art prices (Appendix C, Table C.2), the time trend is not significant in any case and the estimates of d are significantly higher than 1 in almost all cases, the only two exceptions being the global indices (USA, EUR) for which the estimates of d are 0.60 and 0.47 respectively, both implying mean reversion.

Next, we consider the results based on the assumption of autocorrelated errors modelled as in Bloomfield (1973). Table A.3, B.3, and C.3 present the evidence for wine and stamps, diamonds, and art prices respectively, and are structured in the same way as for the case of white noise disturbances. As can be seen, the findings for fine wines (Tables A.3-A.4) are consistent with the previous ones for the white noise case (Table A.2): for all four indices the time trend is found to be insignificant and d is estimated to be significantly higher than 1, which indicates lack of mean reversion.

For diamonds (Tables B.3 and B.4) the time trend is found to be statistically significant (and negative) in 6 out of the 13 cases examined, and mean reversion now occurs in all cases in comparison to 10 out of 13 under the assumption of white noise errors (Table B.2). Nevertheless, the series are very persistent, with the values of d ranging from 0.58 (Diamonds-0.3 Carat Fine Index - PRICE INDEX) to 0.93 (Polished Prices Diamond Index - PRICE INDEX).

Finally, for art prices (Tables C.3 and C.4), the time trend is significant for 8 out of the 13 series investigated (more specifically, positive in 5 cases and negative in 3). Mean reversion (d < 1) is found in 10 out of the 13 cases, and the I (0) hypothesis cannot be rejected for Old Masters and Contemporary (with d = -0.07). Thus, most of these series are highly persistent, especially in the case of fine wines and some of the diamonds and art prices.

To sum up, the above results imply that the passion investment market is far from being efficient. The only series for which some degree of efficiency is found is Polished Prices Diamond Index, for which there is evidence of randomness. All other price series for various types of passion investment (wine, diamond, art, stamp) have long-memory properties. These range from high persistence (as in the case of Wine prices) to anti-persistence (as for majority of the Diamonds indices). These findings partially confirm those of Bouri and Roubaud (2016), Le Fur (2020) and Kumar (2021), whilst they are in contrast to those by Chong et al. (2012), as they provide strong evidence of anti-persistence in the diamonds (ranging from 0.3 to 1 Carat) price indices. The implication of these results is that most passion asset prices are predictable, and that therefore it might be possible to design profitable trading strategies. Persistence analysis is a useful tool to obtain such information.

## 5 Conclusions

This paper explores persistence in the passion investment market. More specifically, it uses R/S analysis (both static and dynamic) and fractional integration techniques to analyse persistence of the following asset prices at the daily, monthly and quarterly frequency: 3 fine wine price indices, 10 diamond price indices, 15 art price indices, and 1 stamp price index. The results can be summarised as follows. Wine prices are found to be highly persistent, whilst stamp prices appear to be less persistent, though they can still be characterised as a long-memory process; as for diamond prices, they can be persistent (Diamonds & Gems), anti-persistent (Diamonds Carat indices) or even random (Polished Prices Diamond Index). The dynamic R/S analysis also shows that persistence is time-varying and tends to fluctuate around the average. These findings can be explained by the different degree of liquidity of the assets examined.

In the majority of cases the evidence appears to contradict the Efficient Market Hypothesis: persistence implies predictability, and anti-persistence more frequent mean reversion than in the case of random series, and in fact in both cases we show that an autocorrelation structure is present in those series. These findings might not be entirely surprising if one considers the fact that “passion” is a key driver of this type of investment in addition to standard reasons such as portfolio diversification, etc. Our analysis, therefore, confirms that the Efficient Market Hypothesis does not hold in this case, namely returns in the passion investment market are not random and instead are predictable. As a result, abnormal profits can be made using trading or investment strategies based on appropriate price forecasting models.

It should be mentioned that the results of both the R/S and fractional integration provide information about the degree of persistence but not about its causes. However, it is plausible to think that differences in data frequencies (daily, monthly, quarterly) might be an important factor as there is evidence suggesting that daily series tend to be closer to randomness, whilst monthly ones are generally more persistent and high-frequency ones are in some cases anti-persistent. Liquidity, trading volumes, and information asymmetries might also play a role. These are interesting issues that could be investigated in subsequent papers.

It is noteworthy that the static values of the Hurst exponent are a snapshot of the current situation, which might evolve over time as confirmed by the dynamic analysis showing that persistence is unstable. Also, given the presence of some possible biases in R/S analysis (see (Taqqu et al., 1995; Lo, 1991) future work could obtain additional evidence using other approaches such as the DFA and DMA methods (Jiang et al., 2019). In addition, other non-linear models such as those based on Chebyshev polynomials in time (Cuestas and Gil-Alana, 2016), Fourier functions in time (Gil-Alana and Yaya, 2021) or even neural networks (Yaya et al., 2021), all them still in the context of long memory or fractional integration, could be estimated using the current or other relevant datasets. This is beyond the scope of the present study and is also left for future work.

## Declarations

### Author contribution statement

Guglielmo Maria Caporale; Luis Gil-Alana; Alex Plastun; Ahniia Havrilyna: Conceived and designed the experiments; Performed the experiments; Analyzed and interpreted the data; Contributed reagents, materials, analysis tools or data; Wrote the paper.

### Funding statement

Alex Plastun was supported by Ministry of Education and Science of Ukraine (0121U100473).

### Data availability statement

Data will be made available on request.

### Declaration of interest's statement

The authors declare no competing interest.

### Additional information

No additional information is available for this paper.

## Figures and Tables

**Table A.1:** Estimates of d: White noise errors

Wines and stamps			
i) Wines			
Series	No deterministic terms	An intercept	An intercept and a linear time trend
Liv-ex Bordeaux 500 Index	1.11 (1.02, 1.19)	**1.49 (1.39, 1.63)**	1.49 (1.38, 1.63)
Liv-ex Fine Wine 100 Index	1.27 (1.17, 1.59)	**1.52 (1.42, 1.63)**	1.52 (1.42 1.63)
Liv-ex Fine Wine Investables Index	1.48 (1.39, 1.59)	**1.52 (1.43, 1.63)**	1.52 (1.43, 1.63)
ii) Stamps			
STANLEY GIBBONS GROUP	1.08 (1.04, 1.11)	**1.18 (1.14, 1.21)**	1.18 (1.14, 1.21)

In bold the selected specification for each series according to the statistical significance of the deterministic terms. In parenthesis the 95% confidence intervals.

**Table A.2:** Estimated coefficients in Table A.1

Wines and stamps			
i) Wines			
Series	d (95% conf. intv.)	Intercept (t-value)	Trend (t-value)
Liv-ex Bordeaux 500 Index	1.49 (1.39, 1.63)	93.056 (22.70)	---
Liv-ex Fine Wine 100 Index	1.52 (1.42, 1.63)	100.495 (37.36)	---
Liv-ex Fine Wine Investables Index	1.52 (1.43, 1.63)	20.509 (6.52)	---
ii) Stamps			
STANLEY GIBBONS GROUP	1.18 (1.14, 1.21)	111.988 (64.53)	----

The values in parenthesis in column 2 are the 95% confidence intervals, while those in columns 3 and 4 are the t-statistics for the coefficients on the constant and the time trend respectively.

**Table A.3:** Estimates of d: *Bloomfield autocorrelated* errors

Wines and stamps			
i) Wines			
Series	No deterministic terms	An intercept	An intercept and a linear time trend
Liv-ex Bordeaux 500 Index	0.96 (0.81, 1.15)	**1.37 (1.20, 1.58)**	1.35 (1.20, 1.58)
Liv-ex Fine Wine 100 Index	0.97 (0.83, 1.13)	**1.32 (1.17, 1.55)**	1.32 (1.17, 1.54)
Liv-ex Fine Wine Investables Index	0.99 (0.80, 1.13)	**1.29 (1.19, 1.44)**	1.29 (1.18, 1.44)
ii) Stamps			
STANLEY GIBBONS GROUP	1.01 (0.96, 1.06)	**1.03 (0.99, 1.07)**	1.03 (0.99, 1.07)

In bold the selected specification for each series according to the statistical significance of the coefficients on the deterministic terms. In red evidence of mean reversion at the 5% level.

**Table A.4:** Estimated coefficients in Table A.3

Wines and stamps			
i) Wines			
Series	d (95% conf. intv.)	Intercept (t-value)	Trend (t-value)
Liv-ex Bordeaux 500 Index	1.37 (1.20, 1.58)	4.608 (344.81)	---
Liv-ex Fine Wine 100 Index	1.32 (1.17, 1.55)	4.534 (233.86)	---
Liv-ex Fine Wine Investables Index	1.29 (1.19, 1.44)	3.020 (123.17)	---
ii) Stamps			
STANLEY GIBBONS GROUP	1.03 (0.99, 1.07)	4.718 (140.69)	---

The values in parenthesis in column 2 are the 95% confidence intervals, while those in columns 3 and 4 are the t-statistics for the coefficients on the constant and the time trend respectively.

**Table B.1:** Estimates of d: White noise errors

Diamonds			
Series	No deterministic terms	An intercept	An intercept and a linear time trend
CCARBNS-DS Diamonds & Gems - PRICE INDEX	1.00 (0.98, 1.02)	**1.02 (0.99, 1.05)**	1.02 (0.99, 1.05)
NORCS-DS Diamonds & Gems - PRICE INDEX	1.00 (0.98, 1.02)	**1.02 (0.99, 1.05)**	1.02 (0.99, 1.05)
WORLD-DS Diamonds & Gems - PRICE INDEX	1.00 (0.98, 1.02)	**1.03 (1.01, 1.06)**	1.03 (1.01, 1.06)
Diamonds-1 Carat Commercial Index - PRICE INDEX	0.97 (0.95, 1.00)	0.61 (0.59, 0.63)	**0.61 (0.59, 0.63)**
Diamonds-1 Carat Mixed Index - PRICE INDEX	0.99 (0.96, 1.02)	0.78 (0.75, 0.81)	**0.78 (0.75, 0.81)**
Diamonds-0.3 Carat Mixed Index - PRICE INDEX	1.00 (0.97, 1.03)	**0.94 (0.91, 0.97)**	0.94 (0.91, 0.97)
Diamonds-1 Carat Fine Index - PRICE INDEX	0.96 (0.94, 0.99)	0.56 (0.55, 0.58)	**0.56 (0.54, 0.58)**
Diamonds-0.3 Carat Commercial Index - PRICE INDEX	0.99 (0.97, 1.02)	**0.78 (0.76, 0.80)**	0.78 (0.75, 0.80)
Diamonds-0.3 Carat Fine Index - PRICE INDEX	0.97 (0.94, 1.00)	0.60 (0.58, 0.62)	**0.60 (0.58, 0.62)**
Diamonds-0.5 Carat Commercial Index - PRICE INDEX	0.99 (0.96, 1.01)	0.68 (0.66, 0.70)	**0.68 (0.66, 0.70)**
Diamonds-0.5 Carat Fine Index - PRICE INDEX	0.97 (0.94, 1.00)	0.58 (0.56, 0.61)	**0.58 (0.56, 0.61)**
Diamonds-0.5 Carat Mixed Index - PRICE INDEX	1.00 (0.97, 1.03)	**0.87 (0.84, 0.89)**	0.87 (0.84, 0.89)
Polished Prices Diamond Index - PRICE INDEX	1.00 (0.97, 1.03)	**0.90 (0.88, 0.92)**	0.90 (0.88, 0.92)

In bold the selected specification for each series according to the statistical significance of the coefficients on the deterministic terms. In red evidence of mean reversion at the 5% level.

**Table B.2:** Estimated coefficients in Table B.1

Diamonds			
Series	d (95% conf. intv.)	Intercept (t-value)	Trend (t-value)
CCARBNS-DS Diamonds & Gems - PRICE INDEX	**1.02 (0.99, 1.05)**	8.5557 (273.88)	---
NORCS-DS Diamonds & Gems - PRICE INDEX	**1.02 (0.99, 1.05)**	8.5565 (271.18)	---
WORLD-DS Diamonds & Gems - PRICE INDEX	**1.03 (1.01, 1.06)**	8.5988 (314.30)	---
Diamonds-1 Carat Commercial Index - PRICE INDEX	**0.61 (0.59, 0.63)**	4.8323 (243.27)	-0.00003 (-4.03)
Diamonds-1 Carat Mixed Index - PRICE INDEX	**0.78 (0.75, 0.81)**	5.1473 (259.15)	-0.00023 (-2.08)
Diamonds-0.3 Carat Mixed Index - PRICE INDEX	**0.94 (0.91, 0.97)**	4.7517 (581.66)	---
Diamonds-1 Carat Fine Index - PRICE INDEX	**0.56 (0.54, 0.58)**	4.9088 (229.20)	-0.00004 (-2.46)
Diamonds-0.3 Carat Commercial Index - PRICE INDEX	**0.78 (0.76, 0.80)**	4.9011 (306.45)	---
Diamonds-0.3 Carat Fine Index - PRICE INDEX	**0.60 (0.58, 0.62)**	4.8731 (205.96)	-0.00008 (-3.52)
Diamonds-0.5 Carat Commercial Index - PRICE INDEX	**0.68 (0.66, 0.70)**	4.8436 (247.36)	-0.00006 (-1.92)
Diamonds-0.5 Carat Fine Index - PRICE INDEX	**0.58 (0.56, 0.61)**	4.8963 (232.06)	-0.00008 (-4.03)
Diamonds-0.5 Carat Mixed Index - PRICE INDEX	**0.87 (0.84, 0.89)**	4.6348 (470.04)	---
Polished Prices Diamond Index - PRICE INDEX	**0.90 (0.88, 0.92)**	8.5980 (314.30)	---

The values in parenthesis in column 2 are the 95% confidence intervals, while those in columns 3 and 4 are the t-statistics for the coefficients on the constant and the time trend respectively.

**Table B.3:** Estimates of d: *Bloomfield autocorrelated* errors

Diamonds			
Series	No deterministic terms	An intercept	An intercept and a linear time trend
CCARBNS-DS Diamonds & Gems - PRICE INDEX	0.98 (0.95, 1.03)	**0.86 (0.82, 0.91)**	0.86 (0.82, 0.91)
NORCS-DS Diamonds & Gems - PRICE INDEX	0.98 (0.95, 1.03)	**0.88 (0.83, 0.91)**	0.88 (0.83, 0.91)
WORLD-DS Diamonds & Gems - PRICE INDEX	0.99 (0.95, 1.03)	**0.88 (0.84, 0.92)**	0.88 (0.84, 0.92)
Diamonds-1 Carat Commercial Index - PRICE INDEX	1.00 (0.94, 1.05)	0.64 (0.62, 0.67)	**0.63 (0.61, 0.66)**
Diamonds-1 Carat Mixed Index - PRICE INDEX	1.00 (0.94, 1.05)	0.81 (0.78, 0.84)	**0.80 (0.77, 0.84)**
Diamonds-0.3 Carat Mixed Index - PRICE INDEX	0.99 (0.95, 1.04)	**0.87 (0.82, 0.90)**	0.87 (0.81, 0.90)
Diamonds-1 Carat Fine Index - PRICE INDEX	0.98 (0.95, 1.03)	0.63 (0.60, 0.65)	**0.63 (0.60, 0.65)**
Diamonds-0.3 Carat Commercial Index - PRICE INDEX	0.99 (0.95, 1.04)	**0.75 (0.72, 0.79)**	0.75 (0.72, 0.79)
Diamonds-0.3 Carat Fine Index - PRICE INDEX	0.98 (0.94, 1.03)	0.59 (0.55, 0.61)	**0.58 (0.55, 0.61)**
Diamonds-0.5 Carat Commercial Index - PRICE INDEX	0.97 (0.93, 1.02)	0.66 (0.64, 0.69)	**0.66 (0.64, 0.69)**
Diamonds-0.5 Carat Fine Index - PRICE INDEX	0.96 (0.92, 1.00)	0.59 (0.57, 0.61)	**0.59 (0.57, 0.61)**
Diamonds-0.5 Carat Mixed Index - PRICE INDEX	0.99 (0.95, 1.03)	**0.88 (0.84, 0.92)**	0.88 (0.84, 0.92)
Polished Prices Diamond Index - PRICE INDEX	1.00 (0.96, 1.04)	**0.93 (0.90, 0.95)**	0.93 (0.90, 0.95)

In bold the selected specification for each series according to the statistical significance of the coefficients on the deterministic terms. In red evidence of mean reversion at the 5% level.

**Table B.4:** Estimated coefficients in Table B.3

Diamonds			
Series	d (95% conf. intv.)	Intercept (t-value)	Trend (t-value)
CCARBNS-DS Diamonds & Gems - PRICE INDEX	**0.86 (0.82, 0.91)**	7.7202 (319.49)	---
NORCS-DS Diamonds & Gems - PRICE INDEX	**0.88 (0.83, 0.91)**	7.7199 (311.76)	---
WORLD-DS Diamonds & Gems - PRICE INDEX	**0.88 (0.84, 0.92)**	7.0521 (306.46)	---
Diamonds-1 Carat Commercial Index - PRICE INDEX	**0.63 (0.61, 0.66)**	**4.8404 (256.73)**	-0.00005 (-2.11)
Diamonds-1 Carat Mixed Index - PRICE INDEX	**0.80 (0.77, 0.84)**	**5.0516 (306.08)**	-0.00013 (-2.01)
Diamonds-0.3 Carat Mixed Index - PRICE INDEX	**0.87 (0.82, 0.90)**	4.7584 (548.97)	---
Diamonds-1 Carat Fine Index - PRICE INDEX	**0.63 (0.60, 0.65)**	**4.8977 (207.64)**	-0.00005 (-1.64)
Diamonds-0.3 Carat Commercial Index - PRICE INDEX	**0.75 (0.72, 0.79)**	4.8997 (293.77)	---
Diamonds-0.3 Carat Fine Index - PRICE INDEX	**0.58 (0.55, 0.61)**	4.8778 (201.03)	-0.00009 (-3.69)
Diamonds-0.5 Carat Commercial Index - PRICE INDEX	**0.66 (0.64, 0.69)**	4.8419 (238.06)	-0.00006 (-1.92)
Diamonds-0.5 Carat Fine Index - PRICE INDEX	**0.59 (0.57, 0.61)**	**4.9008 (213.31)**	-0.00010 (-3.98)
Diamonds-0.5 Carat Mixed Index - PRICE INDEX	**0.88 (0.84, 0.92)**	4.6347 (437.32)	---
Polished Prices Diamond Index - PRICE INDEX	**0.93 (0.90, 0.95)**	4.7251 (947.21)	---

The values in parenthesis in column 2 are the 95% confidence intervals, while those in columns 3 and 4 are the t-statistics for the coefficients on the constant and the time trend respectively.

**Table C.1:** Estimates of d: White noise errors

Artprices índices			
Series	No deterministic terms	An intercept	An intercept and a linear time trend
Global Index (USD)	0.68 (0.57, 0.81)	**0.60 (0.52, 0.72)**	0.61 (0.53, 0.73)
Global Index (EUR)	0.66 (0.55, 0.79)	**0.47 (0.39, 0.59)**	0.49 (0.40, 0.60)
Painting	1.21 (1.06, 1.43)	**1.52 (1.28, 1.95)**	1.52 (1.28, 1.96)
Sculpture	1.10 (0.96, 1.33)	**1.53 (1.23, 2.01)**	1.53 (1.23, 2.04)
Photography	1.03 (0.84, 1.37)	**1.02 (0.79, 1.52)**	1.02 (0.81, 1.52)
Drawing	1.18 (0.94, 1.53)	**1.39 (1.03, 2.13)**	1.39 (1.03, 2.16)
Print	1.45 (1.21, 1.78)	**1.80 (1.42, 2.35)**	1.80 (1.41, 2.32)
Old Masters	0.94 (0.65, 1.49)	**0.79 (0.48, 1.45)**	0.79 (0.46, 1.45)
19th Century	1.15 (0.97, 1.44)	**1.28 (0.97, 1.87)**	1.28 (0.96, 1.88)
Modern Art	1.02 (0.88, 1.25)	**1.27 (1.03, 1.81)**	1.27 (1.03, 1.81)
Post-War	1.56 (1.30, 1.91)	**1.55 (1.29, 1.98)**	1.53 (1.28, 1.95)
Contemporary	0.87 (0.63, 1.24)	**0.88 (0.63, 1.45)**	0.88 (0.64, 1.46)
USA (USD)	1.20 (1.06, 1.43)	**1.52 (1.28, 1.95)**	1.51 (1.28, 1.96)
UK (GBP)	1.06 (0.94, 1.26)	**1.11 (0.98, 1.35)**	1.11 (0.99, 1.33)
France (EUR)	1.12 (0.99, 1.34)	**1.28 (1.10, 1.64)**	1.27 (1.10, 1.60)

In bold the selected specification for each series according to the statistical significance of the coefficients on the deterministic terms. In red evidence of mean reversion at the 5% level.

**Table C.2:** Estimated coefficients in Table C.1

Artprices índices			
Series	No deterministic terms	An intercept	An intercept and a linear time trend
Global Index (USD)	0.60 (0.52, 0.72)	114.322 (6.83)	---
Global Index (EUR)	0.47 (0.39, 0.59)	123.751 (12.08)	---
Painting	1.52 (1.28, 1.95)	96.996 (16.61)	---
Sculpture	1.53 (1.23, 2.01)	98.934 (19.32)	---
Photography	1.02 (0.79, 1.52)	99.985 (6.93)	---
Drawing	1.39 (1.03, 2.13)	98.691 (6.89)	---
Print	1.80 (1.42, 2.35)	92.487 (9.92)	---
Old Masters	0.79 (0.48, 1.45)	111.054 (3.99)	---
19th Century	1.28 (0.97, 1.87)	97.396 (13.43)	---
Modern Art	1.27 (1.03, 1.81)	101.858 (11.78)	---
Post-War	1.55 (1.29, 1.98)	89.494 (4.06)	---
Contemporary	0.88 (0.64, 1.46)	107.172 (2.72)	---
USA (USD)	1.52 (1.28, 1.95)	97.032 (16.56)	---
UK (GBP)	1.11 (0.98, 1.35)	98.956 (17.24)	---
France (EUR)	1.28 (1.10, 1.64)	97.343 (19.72)	---

The values in parenthesis in column 2 are the 95% confidence intervals, while those in columns 3 and 4, are the t-statistics for the coefficients on the constant and time trend respectively.

**Table C.3:** Estimates of d: *Bloomfield autocorrelated* errors

Art prices indices			
Series	No deterministic terms	An intercept	An intercept and a linear time trend
Global Index (USD)	0.95 (0.71, 1.26)	**0.93 (0.75, 1.22)**	0.94 (0.77, 1.21)
Global Index (EUR)	0.93 (0.70, 1.25)	**0.89 (0.65, 1.25)**	0.89 (0.70, 1.24)
Painting	0.91 (0.69, 1.23)	**0.86 (0.75, 1.02)**	0.86 (0.75, 1.02)
Sculpture	0.90 (0.68, 1.21)	**0.65 (0.54, 0.84)**	0.65 (0.51, 0.84)
Photography	0.89 (0.67, 1.18)	0.41 (0.31, 0.53)	**0.34 (0.21, 0.47)**
Drawing	0.90 (0.67, 1.27)	0.42 (0.31, 0.54)	**0.16 (0.01, 0.40)**
Print	0.93 (0.69, 1.23)	0.57 (0.43, 0.75)	**0.55 (0.42, 0.74)**
Old Masters	0.67 (0.40, 0.95)	0.03 (-0.10, 0.21)	**-0.07 (-0.22, 0.10)**
19th Century	0.97 (0.83, 1.13)	1.32 (1.17, 1.55)	**1.32 (1.17, 1.54)**
Modern Art	0.87 (0.67, 1.16)	**0.43 (0.28, 0.61)**	0.41 (0.25, 0.59)
Post-War	0.88 (0.66, 1.24)	0.83 (0.74, 0.98)	**0.81 (0.71, 0.95)**
Contemporary	0.97 (0.73, 1.32)	0.31 (0.21, 0.41)	**-0.05 (-0.19, 0.13)**
USA (USD)	0.91 (0.69 1.23)	**0.86 (0.75, 1.02)**	0.86 (0.75, 1.02)
UK (GBP)	0.92 (0.68 1.22)	0.77 (0.68 0.89)	**0.75 (0.65 0.88)**
France (EUR)	0.91 (0.70 1.21)	**0.80 (0.69 0.94)**	0.80 (0.69 0.94)

In bold the selected specification for each series according to the statistical significance of the coefficients on the deterministic terms. In red evidence of mean reversion at the 5% level.

**Table C.4:** Estimated coefficients in Table C.3

Art prices indices			
Series	No deterministic terms	An intercept	An intercept and a linear time trend
Global Index (USD)	0.93 (0.75, 1.22)	4.610 (38.85)	---
Global Index (EUR)	0.89 (0.65, 1.25)	4.620 (43.69)	---
Painting	0.86 (0.75, 1.02)	4.626 (136.26)	---
Sculpture	0.65 (0.54, 0.84)	4.645 (150.31)	---
Photography	0.34 (0.21, 0.47)	4.771 (97.30)	0.0059 (6.75)
Drawing	0.16 (0.01, 0.40)	4.764 (159.08)	0.0062 (11.79)
Print	0.55 (0.42, 0.74)	4.744 (84.72)	0.0088 (6.89)
Old Masters	-0.07 (-0.22, 0.10)	4.870 (110.75)	-0.0072 (-8.65)
19th Century	1.32 (1.17, 1.54)	4.754 (114.69)	-0.0027 (-3.19)
Modern Art	0.43 (0.28, 0.61)	4.654 (169.23)	---
Post-War	0.81 (0.71, 0.95)	4.657 (68.67)	0.0155 (4.67)
Contemporary	-0.05 (-0.19, 0.13)	4.994 (126.83)	0.0141 (19.24)
USA (USD)	0.86 (0.75, 1.02)	4.619 (136.26)	---
UK (GBP)	0.75 (0.65 0.88)	4.638 (150.81)	0.0052 (4.31)
France (EUR)	0.80 (0.69 0.94)	4.649 (143.95)	---

Values in parenthesis in column 2 are 95% confidence intervals, while in columns 3 and 4, they are t-values for the deterministic terms (constant, column 3; time trend, column 4). In red evidence of mean reversion at the 5% level.

## Appendix A

Fractional integration analysis results for the case of wines and stamps.

## Appendix B

Fractional integration analysis results for the case of diamonds.
